# PRODUCING “QUALITY”: ASSISTED LIVING ADMINISTRATORS’ EXPERIENCE OF THE LICENSING AND SURVEY PROCESS IN OREGON

**DOI:** 10.1093/geroni/igae098.2207

**Published:** 2024-12-31

**Authors:** Ozcan Tunalilar, Jacklyn Kohon, Paula Carder

**Affiliations:** Portland State University, Portland, Oregon, United States; Portland State University, Portland, Oregon, United States; OHSU-PSU School of Public Health, Portland, Oregon, United States

## Abstract

Assisted living (AL) communities in the United States are home to a steadily growing population of over 900,000 individuals and provide assistance with daily activities, medical treatments, and social and recreational activities in a congregate residence. States license AL and oversee regulatory compliance. Studies have examined states’ regulations in terms of stringency and specificity, though they did not address survey processes, especially administrators’ perspective. We conducted semi-structured interviews with 37 AL administrators in Oregon to examine AL administrators’ experiences of the licensing and survey process. Using thematic analysis, we identified three major themes: emotional reactions, practical considerations, and relational assessments. First, administrators expressed feelings of high-level stress related to the survey process. Second, administrators discussed drawing on multiple practical approaches to become “survey ready,” including reviewing prior survey results, having support during a new administrator’s first survey process, and setting up other types of partner or mentor support systems for administrators. Third, administrators defined the survey process through their relationship with surveyors, in terms of tone (adversarial vs. collaborative), hierarchical/power structure (“negotiating the evidence”), and the extent of collaboration (“surveyor as mentor”). We interpret these findings using elements from the Resource Dependency Theory by situating “substantial compliance” as a vital resource for the successful operation of AL and regulatory authorities as key resource providers for AL administrators. These results also emphasize a potential mismatch between how AL administrators view their setting and how regulators do.

